# Significantly Improved Electrical Properties of Crosslinked Polyethylene Modified by UV-Initiated Grafting MAH

**DOI:** 10.3390/polym12010062

**Published:** 2020-01-01

**Authors:** Xin-Dong Zhao, Hong Zhao, Wei-Feng Sun

**Affiliations:** Key Laboratory of Engineering Dielectrics and Its Application, Ministry of Education, School of Electrical and Electronic Engineering, Harbin University of Science and Technology, Harbin 150080, China; xindong_hrbust@126.com

**Keywords:** crosslinked polyethylene, maleic-anhydride, ultraviolet irradiation, electrical conductance, dielectric breakdown strength

## Abstract

Direct current (DC) electrical performances of crosslinked polyethylene (XLPE) have been evidently improved by developing graft modification technique with ultraviolet (UV) photon-initiation. Maleic anhydride (MAH) molecules with characteristic cyclic anhydride were successfully grafted to polyethylene molecules under UV irradiation, which can be efficiently realized in industrial cable production. The complying laws of electrical current varying with electric field and the Weibull statistics of dielectric breakdown strength at altered temperature for cable operation were analyzed to study the underlying mechanism of improving electrical insulation performances. Compared with pure XLPE, the appreciably decreased electrical conductivity and enhanced breakdown strength were achieved in XLPE-graft-MAH. The critical electric fields of the electrical conduction altering from ohm conductance to trap-limited mechanism significantly decrease with the increased testing temperature, which, however, can be remarkably raised by grafting MAH. At elevated temperatures, the dominant carrier transport mechanism of pure XLPE alters from Poole–Frenkel effect to Schottky injection, while and XLPE-graft-MAH materials persist in the electrical conductance dominated by Poole–Frenkel effect. The polar group of grafted MAH renders deep traps for charge carriers in XLPE-graft-MAH, and accordingly elevate the charge injection barrier and reduce charge mobility, resulting in the suppression of DC electrical conductance and the remarkable amelioration of insulation strength. The well agreement of experimental results with the quantum mechanics calculations suggests a prospective strategy of UV initiation for polar-molecule-grafting modification in the development of high-voltage DC cable materials.

## 1. Introduction

Although the clean and renewable electric power generations have become the focuses for future power system in recent years [1], these new decentralized and miniaturized power sources, such as solar or wind energy generation which are connected into the power grid using alternating current (AC) transmission technology, are generally poor in terms of economics and environmental protection due to their long distance from power centers. At present, high voltage direct current (HVDC) transmission technology on account of voltage source converters (VSCs) has been significantly developed to realize power transmission of large capacity over long distances [2]. As the pivotal equipment to constitute DC power grid, crosslinked polyethylene (XLPE) insulated cables with higher insulation performances are obligatory to guarantee secure and stable operation of a power transmission system [3,4,5]. Despite the high operating temperature, light weight and ease of manufacturing, XLPE insulated HVDC cable is susceptible to charge injection and space charge accumulation under an HVDC electric field, which eventually results in insulation aging and dielectric breakdown caused by electric field distortion. Moreover, the temperature gradient existing in working cables causes the spatially altering electrical conductance in XLPE insulating material, and consequently changes electric field distribution inside the insulated cable which will feedback the electrical conductivity by electric heating. Therefore, it is urgent to develop polyethylene materials with thermostable conductance characteristics.

Nowadays, the primary strategies of developing XLPE insulation materials are represented by the ultra-clean processing of pristine polyethylene, nanocomposite technology and chemical modifications [6]. The polymer dielectric nanocomposites (nanodielectrics) have manifested substantially improved dielectric properties, such as inhibited space charge accumulation and reduced electrical conductance, and enhanced breakdown strength in comparison to neat polymer material, which originates from the numerous deep traps for charge carriers caused by polar molecular groups at nanofiller/matrix interfaces [7,8]. Thomas reported various strategies for XLPE coupled with different nanofillers to enhance the electrical insulation properties for high power transmission, and comprehensively summarized the significance of SiO_2_, Al_2_O_3_, TiO_2_, MgO and BN-based XLPE nanocomposites as potential candidates for various applications in high voltage cables, electrical transistors and thermal insulation [9]. However, the modification efficiency relies greatly on the dispersivity and surface states (impurities and defects) of nanofillers in the polymer matrix, which cannot be facilely controlled due to the infinitely large surface/volume ratio [10,11]. Accordingly, it is urgent to explore a tactical modification routine to circumvent the inevitable faultiness in nanodielectrics. It was reported and revealed in an underlying mechanism that modified electrical properties of polypropylene, such as space charge suppression and breakdown strength improvement with abated conductivity, can be achieved by chemically grafting maleic anhydride [12]. Chemically modifying XLPE by grafting chloroacetic acid allyl ester and the correlated space charge accumulation with a trapping mechanism have been reported to explain the palpable improvement of insulation performances [13]. S.H. Lee suggested that the heteropolar space charge accumulation and electrical conductance are definitely impeded in low density polyethylene (LDPE) by successfully grafting maleic anhydride (MAH) to a molecular chain of polyethylene with general chemical methods [14]. Nevertheless, the normal chemical grafting processes of employing organic peroxide are performed by heated parallel twin screws, in which the grafting reactions of MAH molecules with polyethylene cannot definitely be separated from the crosslinking reactions between polyethylene macro-molecules. Therefore, it is inevitable to introduce cross-linked polyethylene into the grafting products and form the scorched substance of earlier crosslinking, which is acknowledged as “amber color” of self-generated impurity particles, and severely deteriorate the electrical resistance of cable insulation.

To this end, we developed a novel grafting process with ultraviolet (UV) irradiation technique in which LED light source of 365 nm wavelength and photon-initiator are utilized to induce the broken double bonds of MAH and engender radicals on macro-molecule chains of melting polyethylene. Because the macro-molecular movement of polyethylene requires a long relaxation time, while the grafting of small MAH molecules to polyethylene tends to be completed transiently, the MAH-grafted polyethylene materials without gel caused by self-generated impurities can be obtained by accurately controlling the irradiation time and the dose of photon-initiator. According the suggested routine, employing a photo-initiator and UV irradiation, we successfully grafted MAH to polyethylene molecules and eventually prepared the modified XLPE materials (XLPE-g-MAH) with preferable dielectric properties. By means of first-principles calculations, the underlying mechanism of improving insulation performances was also theoretically elucidated by analyzing the electronic bound states in band-gap of XLPE-g-MAH. Beyond the general tests, as carried out at a constant ambient temperature, the electrical conductivity and breakdown strength measurements at variable temperature were accomplished to correlate electronic properties with electrical conductance and dielectric breakdown.

## 2. Experiments and Theoretical Methodology

### 2.1. Material Synthesis

High efficiency of grafting reactions can be gained by firstly preparing the master-batches with high content of grafting MAH to LDPE. The fundamental raw materials in synthesis experiments are employed as follows: LDPE (LD200GH, Sinopec Company Ltd., Beijing, China) as matrix material, MAH (Ruierfeng Chemical Co., Ltd. Guangzhou, China) as graft reactant, dicumyl peroxide (DCP, Nobel Company Ltd., Aksu, China) as the auxiliary crosslinking agent, benzophenone (BP, Jinleiyuan Chemical Co., Ltd., Lianyungang, China) for initiating grafting reaction under UV irradiation and pentaerythritol ester (Irganox1010, Shanyi Plastics Co., Ltd., Dongguan, China) as an antioxidant. In melting blend process of preparing the initial mixtures, the pristine LDPE materials are heated to be melted at the temperature of 120 °C with a stirring speed of 60 rpm in Torque Rheometer (RM200C, Hapro Company Ltd., Harbin, China) for 1 min, and then 3 wt% MAH and 0.2 wt% BP are added, being blended for 3 min and cooled down to room temperature. The obtained uniform mixing materials are eventually cut into granules for testing. In the process of photon-initiated grafting reactions, the prepared mixtures are firstly melted in a plate vulcanizer at 120 °C temperature under the pressure being raised by 5 MPa per 5 min from 0 to 15 MPa, which will transverse through the photon-initiation platform under UV irradiation for 2 s at normal pressure and room temperature in air atmosphere. The MAH-grafted LDPE (LDPE-g-MAH) with modified electrical properties is finally prepared after short-circuit degassing at 80 °C for 48 h in a vacuum oven. The gel contents of LDPE-g-MAH materials prepared with the UV initiating technique are tested by the gel extraction experiment to be lower than 0.01%—remarkably lower than the gel content of chemically-grafted polyethylene materials.

Secondly, the prepared LDPE-g-MAH materials are mixed into LDPE with an appropriate proportion so as to dilute the MAH content to a reasonable level which could effectively inhibit electrical conductance and breakdown. The grafting-modified XLPE material (XLPE-g-MAH) can be eventually achieved after the necessary crosslinking reactions of LDPE-g-MAH material by melt-mixing them with the pure LDPE, DCP auxiliary crosslinker and antioxidant 1010 (with the blending components as listed in Table 1) in torque rheometer at the temperature of 110 °C temperature and the rotate speed of 60 rpm for 5 min. In order to completely accomplish the crosslinking reactions, the mixed materials are further treated under 15 MPa at 175 °C for 1 h in the plate vulcanizer. The melting XLPE-g-MAH materials are solidified by cooling down to ambient temperature at a rate of 25 °C/min under 15 MPa in a cooler. Finally, the prepared samples should be hot-degassed at 80 °C for 48 h in a vacuum oven to eliminate internal stress and remove reaction by-products. The MAH graft concentrations of LDPE-g-MAH master-batches and XLPE-g-MAH materials are measured by chemical titration method.

### 2.2. Characterization and Testing Methods

Employing a Fourier-transform infrared (FT-IR) spectrometer (FT/IR-6100, Jiasco Trading Co., Ltd., Shenyang, China) with the wavenumber range of 500–4000 cm^−1^ scanned in the resolution of 2 cm^−1^, the grafted MAH in modified materials were characterized by infrared transmission spectrum for a film sample in 0.3 mm thickness.

Electrical conductivity was measured by a standard three-electrode system at various temperatures from 25 to 80 °C for the film samples in thickness of 0.2 mm with two evaporated aluminum electrodes on one side as measuring and protective electrodes respectively and one evaporated aluminum electrode on the other side for applying high voltage. After being heated and stabilized at the testing temperature for 60 min, the tested samples are applied with the electric field at each point covering 3–40 kV/mm range for 60 min to measure conductance current. The DC dielectric breakdown strength (DBS) of the circular film samples with a diameter of 80 mm and a thickness of 0.1 mm are tested by asymmetric columnar electrodes (25 mm and 75 mm in diameter for high voltage and ground electrodes respectively), in which the maximum voltage is recorded before the sample breakdown when the applied electric field is raised continuously at a constant speed of 4 kV/s.

### 2.3. Molecular Model and Calculation Methodology

Molecular simulations of symptomatic polyethylene molecule being grafted by MAH (PE-g-MAH) were performed based on the equilibrium C–C and C–H bond lengths of 1.50 Å and 1.10 Å, respectively, with randomly distributed torsion, in which the PE molecule spolymerizing at 30 degrees and being grafted by MAH molecules at the middle position of PE backbone chain are initially constructed by the rotational isomeric state (RIS) model. The structural relaxation of initial polymer configurations were obtained in geometry optimization by total energy functional minimization with conjugated gradient algorithm in first-principles calculations [15], such that the energy change, atomic force and displacement were theoretically evaluated to be lower than 1.0 × 10^−5^ eV/atom, 0.03 eV/Å and 0.001 Å respectively. The electronic structures were calculated for molecular orbitals and electronic density of states to investigate band-edge features and grafting-introduced trap states. The first-principles calculations were performed with all-electron and numerical atom-orbitals, as implemented in DMol3 program of Materials studio 8.0 software package (Accelrys Inc., Materials Studio v8.0.0.843, San Diego, CA, USA), as the detailed methodology adopted in calculations listed in Table 2.

## 3. Results and Discussion

### 3.1. Molecular Structure Characterization

**Figure 1:**
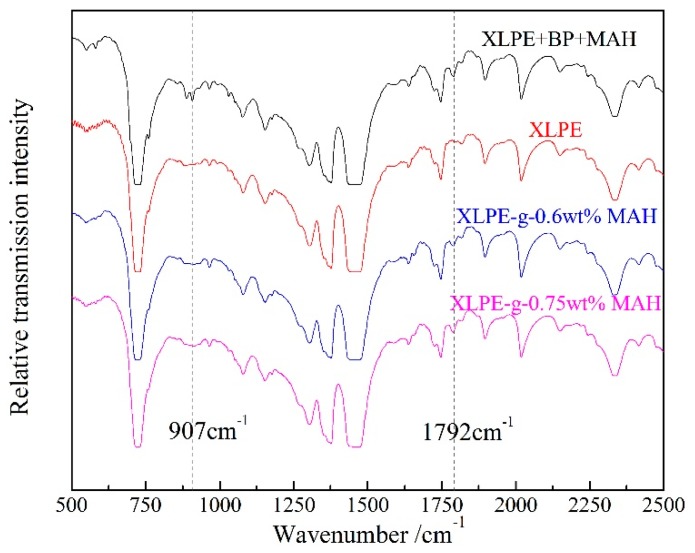
FT-IR transmission spectra of XLPE, XLPE + BP + MAH and XLPE-g-MAH for 0.6 wt% and 0.75 wt% grafting concentrations.

The grafted MAH on the polyethylene molecular chain is characterized by IR spectroscopy in comparison to the homologous mixture sample obtained though the similar preparation process without UV irradiation, as the results show in Figure 1, implying the molecular transformation caused by UV-initiated grafting reaction. The carbonyl (C=O) of MAH molecule can be identified by the stretching vibration peak at 1792 cm^−1^ of IR adsorption. The intensity of the newly appeared C=O peak for XLPE-g-MAH increases with the MAH graft concentration, to the highest value for XLPE-g-0.75 wt%MAH. Especially, the IR absorption peak at 907 cm^−1^ coming from carbon double bond (C=C) of MAH monomer was only observed in homologous mixture of XLPE, BP and MAH (XLPE + BP + MAH) without grafting reaction, and did not arise in the IR spectra of XLPE-g-MAH, showing that the grafted MAH groups are chemically connecting to the molecular chains of XLPE. The FT-IR results reasonably demonstrate that MAH molecules were successfully grafted to XLPE molecules for each graft concentration.

### 3.2. Electrical Conductance

**Figure 2:**
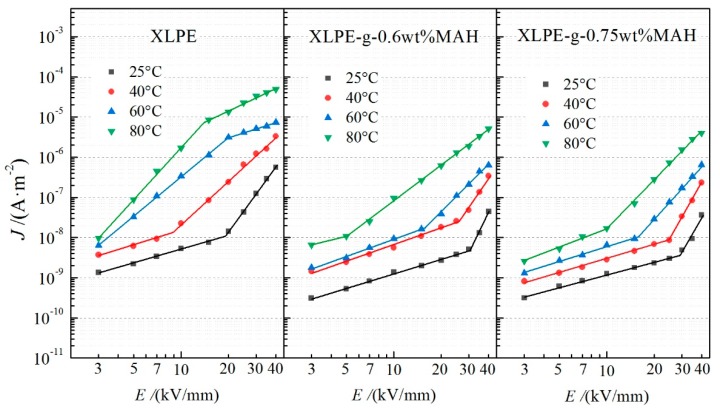
Conductivity properties of *J*–*E* varying curves for XLPE, XLPE-g-0.6 wt%MAH and XLPE-g-0.75 wt%MAH at the individual temperatures from 25 °C to 80 °C.

Electrical conductance behaviors of the XLPE and XLPE-g-MAH materials were tested under the DC electric field varying from 0 to 40 kV/mm at different temperatures, as with the current density-electric field (*J*–*E*) curves shown in Figure 2. For the same temperature and applied electric field, the conductivity of the XLPE-g-MAH samples is observably lower than that of pure XLPE. Under the electric field of 20 kV/mm, the conductance current of pure XLPE increases by three orders of magnitude when the temperature rises from 25 °C to 80 °C. In contrast, the XLPE-g-MAH represents much less temperature dependence of electrical conduction. At the archetypal operating temperature of DC insulated cable (25–80 °C), the electrical conductivity of XLPE-g-MAH shows a remarkably less increment, by one order of magnitude, than pure XLPE as the applied electric field is raised from 3 kV/mm to 40 kV/mm. Although no obvious difference in the conductivity of XLPE-g-MAH materials can be observed below 40 °C, a considerably lower conductivity was acquired by XLPE-g-0.75 wt%MAH than the 0.6 wt% MAH grafting when the temperature increased to higher than 40 °C, suggesting that better thermostable conductance can be achieved by increasing the content of grafting MAH.

Based on the gradients of *J*–*E* curves in logarithmic coordinates, the electrical conductance can be distinguished into ohm region and trap active region, the demarcation point of which represents the critical electric field that the dominant carrier transport altering from ohm conductance to charge trap scattering (space charge limited conductance) [17]. This critical field rapidly decreases as the testing temperature is increased, and even disappears for pure XLPE at the temperatures higher than 40 °C. The *J*–*E* curves of Figure 2 manifest that the grafting MAH causes an observable increment of this critical electric field. Consistently with the mechanism of trap-limited conductance [16], the critical electric field enhancement of XLPE-g-MAH is attributed to the higher barrier that the carriers need to overcome when they are transporting through the densely and uniformly distributed deep traps which have been introduced by grafting MAH.

**Figure 3:**
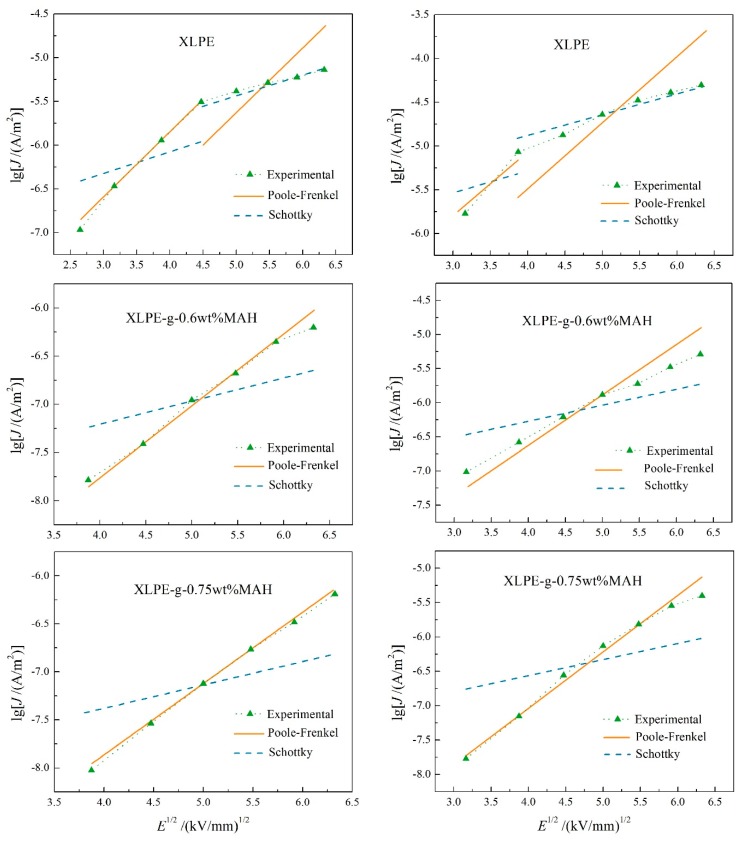
Logarithmic current density versus 1/2 power of electric field and piece-wise fitting lines for pure XLPE (**upper panels**), XLPE-g-0.6 wt%MAH (**middle row panels**) and XLPE-g-0.75 wt%MAH (**bottom panels**) at 60 °C (**left panels**) and 80 °C (**right panels**).

At high temperatures, XLPE represents various electrical conductance mechanisms under high electric field, in which the carrier transports will be dominated by Poole–Frenkel effect or Schottky injection [18]. The conductance characteristics of Figure 2 indicate that the conductance currents of XLPE and XLPE-g-MAH under a high electric field at elevated temperatures are not dominated by space charge limited conductance. It is well known that the characteristic curves (lg*J*–*E*^1/2^) of the conductance currents dominated by Poole–Frenkel effect and Schottky injection show linear variations, the different gradients of which can be used to discriminate the dominant conductance mechanism. Therefore, the lg*J*–*E*^1/2^ curves are plotted with piece-wise linear fittings from the test results of conductance currents under high electric fields at the temperatures of 60 °C and 80 °C, as shown in Figure 3. With the increase of electric field in high field region (non-ohmic region), the conductance mechanism of XLPE changes from Poole–Frenkel effect to Schottky injection at a critical electric field which gradually decreases with the increasing temperature, as shown by the upper panels in Figure 3. In contrast, the electrical conduction of XLPE-g-MAH is always dominated by Poole–Frenkel effect in high field region at the temperature of 60 °C. When the temperature reaches 80 °C, the conductance current of XLPE-g-0.6 wt%MAH originates primarily from the combined contributions of both Poole–Frenkel effect and Schottky injection, as shown by the plotted experimental curves with the gradient being between the slopes of two fitting lines in the middle row panels of Figure 3. In particular, the conduction mechanism of XLPE-g-0.75 wt%MAH is dominated only by Poole–Frenkel effect at both 60 and 80 °C, as shown in the bottom panels of Figure 3. The charge carriers injected from electrodes under high electric field will be captured on the material surface by the fixed and densely distributed deep traps which are introduced by grafting MAH, and thus form an effective electrostatic-shielding layer so as to impede the further Schottky carrier injection from electrodes. It is well known the inevitable physical defects existing in XLPE offer the intrinsic charge traps with shallow levels (≈0.9 eV) which become invalid in capturing carrier at the temperature higher than 60 °C due to thermal excitation. Whereas, the deep level traps (>1.4 eV as discussed in next paragraph) introduced by grafting MAH can retain the capability of capturing electronic carrier at the temperature approaching to 150 °C. Therefore, UV-initiated grafting MAH has effectively inhibit the Schottky injection into XLPE under high electric field at elevated temperatures, which can be further improved by increasing the grafting content.

**Figure 4:**
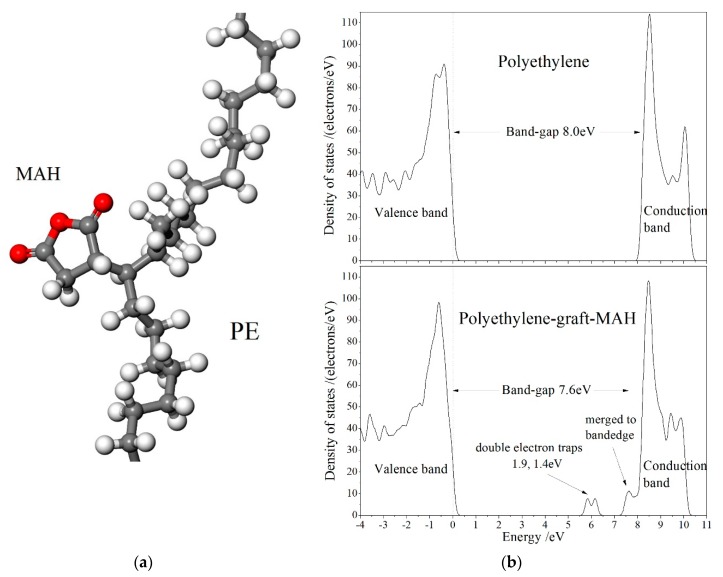
(**a**) Schematics of grafting MAH to polyethylene molecule with gray, white and red spherules identifying carbon, hydrogen and oxygen atoms respectively; (**b**) density of states (DOS) of PE and PE-g-MAH from first-principles calculations, in which the highest occupied molecular orbital (HOMO) level is set as the energetic zero, as indicated by the vertical dashed line.

As shown in Figure 4 for the relaxed (geometrically optimized) model and density of states (DOS) of polyethylene molecule grafted by MAH (PE-g-MAH), two unoccupied electronic bound states are introduced in the band-gap of PE molecules by grafting MAH. These electronic bound states serving as deep traps with level depths of 1.4 eV and 1.9 eV will be densely distributed in XLPE-g-MAH to scatter electronic carriers in high probability, and can form high-temperature-valid electrostatic a shielding layer near cathode to inhibit Schotky injection. Therefore, the higher critical electric field of XLPE-g-MAH than that of XLPE is attributed to the shorter electronic free path and the fewer electronic carriers caused by grafting-introduced deep traps. Meanwhile, the density of states near band-edge of polymer molecules pertains to the probability of carrier transition from between the adjacent levels of electronic states, and thus dominates the carrier mobility without consideration of impurity or defect scattering. Accordingly, very close to conduction band minimum (CBM) of polyethylene, an unoccupied bound state of a little lower energy level is also introduced by the grafted MAH which merged into the conduction band to become a new CBM, resulting in a substantially decreased DOS on the conduction band-edge, as illustrated by Figure 4b. This means the considerably lower carrier mobility was acquired, and thus consistently explains the subdued conductance in XLPE-g-MAH. Based on the electronic structure theory of condensed matter physics, carrier mobility can be reduced from carrier-phonon scattering only when the temperature is being highly raised, eliminating the temperature effect on carrier mobility of XLPE-g-MAH materials at 25–80 °C.

### 3.3. Electric Field Distribution in Cable

**Figure 5:**
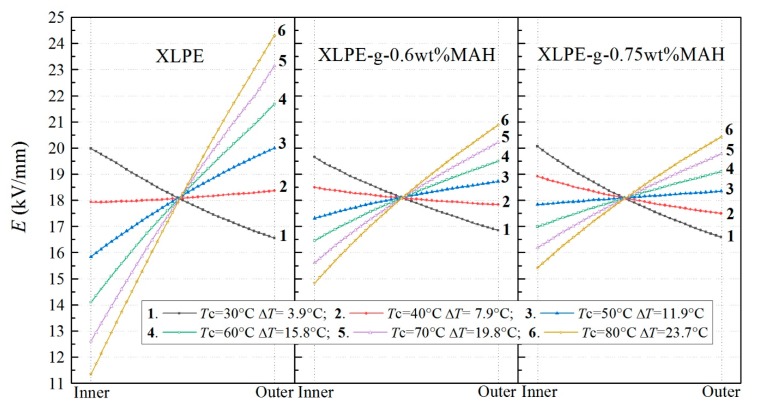
Simulated electric field distributions in insulation layers of a DC cable modeled with XLPE, XLPE-g-0.6 wt%MAH and XLPE-g-0.75 wt%MAH materials respectively.

In order to prevent the electric field on the outside of insulation layer from exceeding the designed upper limit when the insulated cables are fully loaded, the temperature dependence of the electrical conductivity is required to be minimized as much as possible in the design and fabrication of DC insulated cables. Therefore, in order to investigate the influence of the conductance thermostability acquired by XLPE-g-MAH materials on the electric field distribution in insulation layer of HVDC cable, a typical structure of ±525 kV HVDC cable with XLPE or XLPE-g-MAH insulation layer is simulated by finite element numerical schemes of electric-thermal coupling [19]. In our cable model, the insulation thickness is 31 mm, the applied voltage is 525 kV, the ambient temperature is 25 °C, and the conductive cable core temperature is set alternating in the temperature range of 30–80 °C. The temperature difference between the inner and outer shields of insulation layer and electric field distribution in insulation layer were calculated for different core temperatures, as the results show in Figure 5.

For XLPE insulation layer, when the core temperature reaches 40 °C, the electric field inversion occurs with the maximum electric field in XLPE locating at the outer shield, and the temperature difference between the inner and outer shields increases with the increased core temperature, magnifying the change of XLPE conductivity caused by temperature gradient. Therefore, the electric field inversion is more likely to occur in XLPE insulation layer for higher core temperature and maximum electric field reached 24.4 kV/mm for the core temperature of 80 °C. In contrast, the electric field inversion is effectively inhibited in XLPE-g-MAH insulation layer of HVDC cable due the more thermostable conductivity. The maximum electric field of XLPE-g-0.6 wt%MAH insulation layer is limited to the 20.9 kV/mm even when the core temperature rise to 80 °C. Even though the electric field inversion obviously occurred for XLPE-g-0.75 wt%MAH insulation layer when the core temperature just rose to 50 °C, the maximum electric field of 20.4 kV/mm appearing at outer shield is still distinctly lower than that for XLPE insulation layer.

### 3.4. Dielectric Breakdown Strength

**Figure 6:**
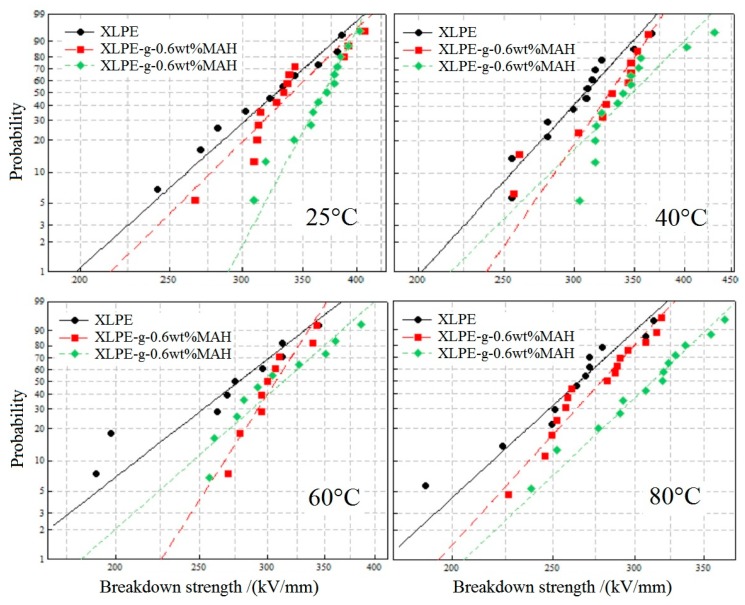
Dielectric breakdown strength (DBS) statistics fitted with two-parameter Weibull distribution for XLPE and XLPE-g-MAH at the temperatures of 25–80 °C.

DBS tests were performed in the temperature range of 25–80 °C for XLPE and XLPE-g-MAH, the results of which were analyzed by Weibull statistics fitted with a two-parameter distribution [20], as per the results provided in Figure 6. The characteristic DBS can be described by the field strength at 63.2% breakdown probability which is directly obtained from the scale and location parameters of the fitted Weibull distribution, as for the results listed in Table 3. The modified XLPE materials by UV-initiated MAH graft represent notable DBS improvement at various temperatures, confirming the deep-trap effect originating from the anhydride group in the grafted MAH. The XLPE-g-MAH materials acquired at least 9.9% higher DBS than the characteristic 63.2% of pure XLPE due to the deep traps introduced by MAH graft, which is consistent with the charge trapping and scattering mechanism in suppressing space charge accumulation and impeding electrical conductance under a DC electric field [21]. Moreover, the MAH grafting concentration will substantially affect the DBS, especially at higher temperatures. The photon-initiated graft modification on DBS was still retained for increasing temperature to 80 °C, as shown in Table 3. Based on the experimental results of electrical conductance and the quantum mechanics calculations, it is reasonable to correlate DBS improvement with the increment of trap level depth and density. These results support the electron trapping model of ameliorating dielectric properties by grafting polar group and suggest that the MAH is the mostly expected graft candidate to improve insulation performances of polymer materials [13]. The tight coupling carbonyl carbon atoms of cyclic anhydride in MAH also essentially account for the enhanced DBS of XLPE-g-MAH.

## 4. Conclusions

Significantly improved insulation performances of MAH-grafted XLPE dielectrics have been achieved by employing a UV initiation technique, and the correlated modification mechanisms were investigated in combination with quantum mechanics calculations. The XLPE-g-MAH materials with almost zero gel content have been synthesized through the successful UV-initiated reactions of grafting MAH to polyethylene molecules, which are characterized by gel extraction experiments and IR spectroscopy. The XLPE-g-MAH materials acquired excellent DC dielectric properties, such as decreased electrical conductivity and enhanced dielectric breakdown strength, especially at elevated temperatures, which are effective at retarding the inversion of electric field distribution caused by the temperature gradient in XLPE insulation layer of HVDC cable. The grafted MAH on polyethylene molecules can introduce electronic bound states at CBM and deep traps in band-gap of polyethylene, which account for the decreasing carrier mobility. The modified insulation performances of XLPE-g-MAH were attributed to the double deep traps introduced by grafting MAH, which can remarkably impede Schottky carrier injection from electrodes by forming electrostatic shielding layer of captured charges near electrodes under high electric fields at elevated temperatures. The present study promises a strategic routine for graft modifications to fulfill high performance insulating materials in HVDC cable productions.

## Figures and Tables

**Table 1:** Mixture components for preparing XLPE and XLPE-g-MAH samples.

Sample	LDPE/wt%	LDPE-g-MAH/wt%	DCP/wt%	1010/wt%
XLPE	97.8	0	2.0	0.3
XLPE-g-0.75 wt%MAH	73.3	24.4	2.0	0.3
XLPE-g-0.6 wt%MAH	78.2	19.6	2.0	0.3

**Table 2:** Schemes and parameters adopted in the first-principles calculations of DMol3.

Electronic Hamiltonian	Scheme	Condition and Parameter
Exchange-correlation energy	Meta-generalized-gradient approximation	M11-L [16]
Integration accuracy		2000 grid points /atom
SCF	Tolerance	1 × 10^−6^ eV/atom
	Multipolar expansion	Octupole
	Charge density mixing	Charge = 0.3, DIIS = 5
Core treatment	All Electron	
Numerical basis set	DNP	Basis file 4.4
Orbital cutoff	Global	5.0 Å

**Table 3:** The characteristic 63.2% DBS of Weibull distribution fitted in a 95% confidence interval at different temperatures (kV/mm).

**Samples**	25 °C	40 °C	60 °C	80 °C
**XLPE**	341.8	319.8	293.4	276.1
**XLPE-g-0.6 wt%MAH**	353.8	337.8	315.4	288.1
**XLPE-g-0.75 wt%MAH**	375.7	362.4	329.7	324.2
